# Using a combination of biomarkers to monitor allograft dysfunction in lung transplant recipients

**DOI:** 10.3389/frtra.2025.1574898

**Published:** 2025-05-08

**Authors:** Zein Kattih, Shambhu Aryal

**Affiliations:** Advanced Lung Disease and Transplant Program, Inova Heart and Vascular Institute, Inova Fairfax Hospital, Falls Church, VA, United States

**Keywords:** cell-free DNA, antibody-mediated rejection, acute cellular rejection, chronic lung allograft dysfunction, lung transplant

## Abstract

Allograft dysfunction is a major limitation of survival in organ transplant recipients including those who have received lung transplantation. Early detection of allograft dysfunction is thus crucial to improve outcomes in these patients. However, there are several causes of allograft dysfunction with allograft infection and rejection being the two important causes. It is often difficult to distinguish between those causes as the presentation can be similar. Allograft rejection, especially antibody-mediated rejection (AMR) and chronic lung allograft dysfunction (CLAD) are often identified too late where progression has already occurred. Biomarkers like anti-HLA antibodies including donor-specific antibodies (DSA), donor-derived cell-free DNA (dd-cfDNA), immune cell function (ICF) assays and next-generation sequencing for microorganisms allow for early identification of allograft dysfunction as well as differentiate rejection from other processes such as infection. This in turn allows for early intervention and, ideally, improved long-term allograft outcomes. Greater evidence exists for these biomarkers in other solid organ transplantations including kidney and heart transplantation, but application to lung transplant recipients is increasing and seems equally promising. In this review, we evaluate existing evidence for using these biomarkers and share our center practice in utilizing a combination of these biomarkers post-transplantation to assess for allograft dysfunction.

## Introduction

Lung transplantation is a life-saving therapy for many individuals with end-stage lung disease. However, the median survival after lung transplantation is only 6.5 years based on the cohort of transplant recipients from 1990 to 2015, far lower than other solid organ transplants including kidney and heart transplantation (1). Allograft dysfunction is a major cause of morbidity and mortality in lung transplant recipients (1). Between 30 days and one year after the transplant, graft failure accounts for 22.7% of deaths (1). Beyond one year, graft failure accounts for 40% of deaths (1). Historically, monitoring for rejection has been primarily accomplished via pulmonary function testing and clinical evaluation. Surveillance bronchoscopies with bronchoalveolar lavage (BAL) and transbronchial biopsies may be performed; however, there is no clear evidence that surveillance bronchoscopy is better for detecting acute rejection in comparison to clinically indicated bronchoscopy (2). In recent years, several biomarkers have become available for the evaluation of allograft function with mounting evidence, even in the absence of clinical features of rejection. These biomarkers can be utilized to quantify the net state of immunosuppression in patients and may help to describe the risk of allograft dysfunction.

Many biomarkers have been developed and evaluated in solid organ transplant recipients. These biomarkers include anti-human leukocyte antibodies (HLA) including those directed against donor organs and thus termed donor-specific antibodies (DSA), non-HLA antibodies, Torque tenovirus (TTV) testing, gene expression profiling (GEP), donor-derived cell-free deoxyribonucleic acid (dd-cfDNA), cell immune monitoring assays, and micro ribonucleic acid (RNA), among others (3–5). Some molecular monitoring tools, including DSA, dd-cfDNA, immune cell function (ICF) assays, TTV testing, tissue transcriptomics, microRNA evaluation from blood and BAL samples, exosomes, and methylation markers, have varying degrees in evidence in lung transplant recipients (6). Biomarkers with the most robust evidence are discussed below.

### Donor-specific antibodies (DSA)

Acute antibody-mediated rejection (AMR) in lung transplantation is defined by the presence of four criteria, namely allograft dysfunction, the presence of DSAs, characteristics histopathologic findings, and deposition of complement factor 4d (C4d) on the capillary endothelium, in addition to ruling out other etiologies of graft dysfunction (7). Peripheral immunologic cells express HLA antigens, which are subdivided into Class I (HLA-A, B, C) and Class II (HLA-DR, DP, DQ) based on their structure and function (7). C4d detection can be performed using two immunopathologic assays-immunofluorescence and immunohistochemistry.

**Figure 1:**
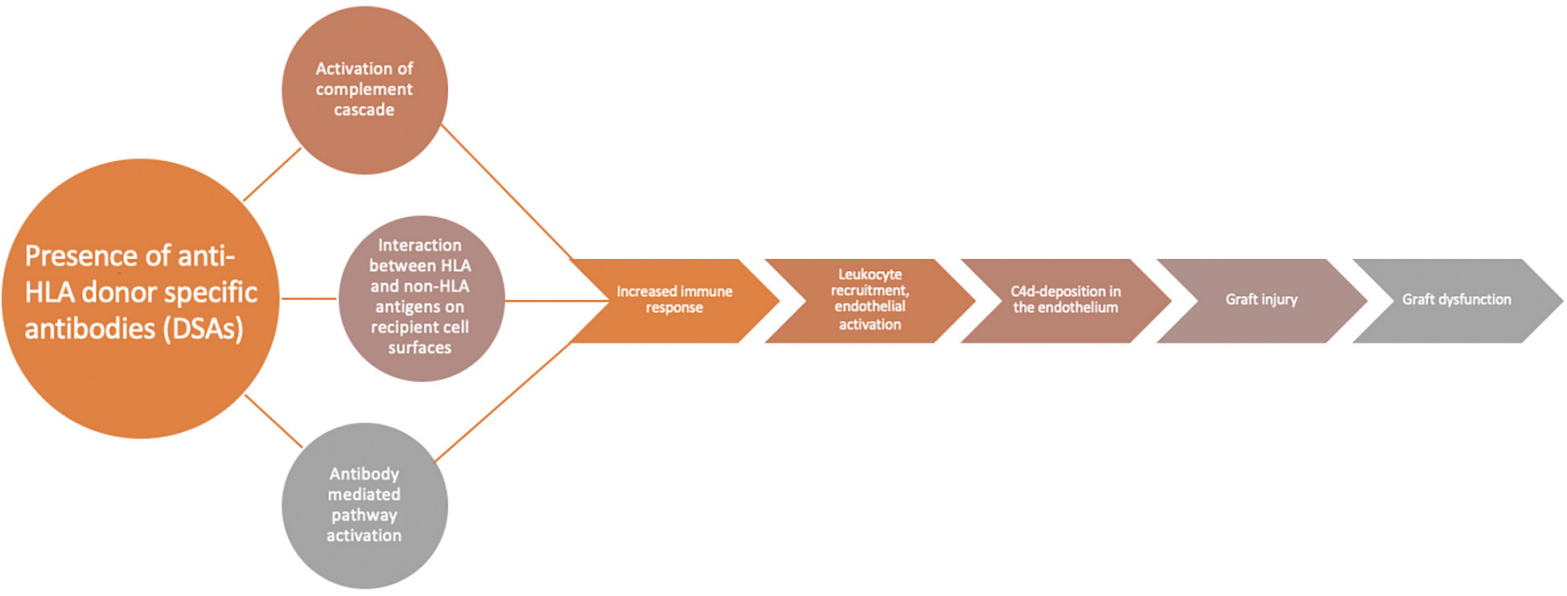
DSAs and AMR. Flow diagram demonstrating the role of DSAs in the development of AMR. The presence of DSAs leads to activation of complement cascade, the interaction between HLA and non-HLA antigens, and antibody-mediated activation which all lead to increased immune response, leukocyte recruitment, endothelial activation, and ultimately result in CD4 deposits in the allograft, graft injury, and graft dysfunction. Adapted with permission from “Diagnostic criteria for AMR” and “Mechanism of AMR” by Shourjo Chakravorty, Shambhu Aryal Adam Cochrane, and Steven D. Nathan licensed under CC-BY 4.0.

DSAs are central to the development of AMR through multiple mechanisms (Figure 1). These molecules can lead to complement activation through the classical pathway and cause an increase in immune response, endothelial cell necrosis and, ultimately, graft damage (8). Interaction between these HLAs and non-HLA antigens on cell surface as well as antibody-mediated pathway activation can lead to natural killer cell activation, further leading to graft damage (8). Leukocyte recruitment and endothelial activation also contribute to AMR (8). Measurement of DSAs has historically been performed utilizing donor-derived peripheral T-lymphocytes as surrogates to detect complement-dependent activations (9, 10). Advances in laboratory techniques now allow detection of DSA through flow cytometry independent of complement fixation (8, 9). Most recently, a bead-based immunoassay platform allows for enhanced sensitivity and specificity in the detection of these antibodies (8, 11). Measurement of circulating HLA antibody titer is important in the surveillance of graft function, especially in combination with evaluation for clinical dysfunction, histologic evidence of AMR, and C4d detection (7). While studies have attempted to categorize patients with AMR based on DSA positivity (12), controversy remains on how to utilize DSA as a sole marker of rejection, especially in clinically stable patients (7).

Donor-specific anti-HLA antibodies contribute to the development of antibody-mediated rejection (AMR) in lung transplant recipients (7, 12) (Table 1), though the assays used to measure DSAs and the intervals at which DSAs are monitored vary from study to study. DSAs have also been associated with CLAD (12–15), especially development of *de novo* HLA-DQ DSA (14). Development of *de novo* DSA has been reported to occur in 13%–61% of lung transplant recipients (13–15). The development of clinical AMR is often a late phenomenon, and poor outcomes of AMR despite treatment is probably due to delayed intervention. As such, preemptive treatment of *de novo* DSA may have a role in lung transplant recipients. In a study of 445 patients, 145 of whom developed *de novo* DSA after transplantation (including re-do transplantation), early treatment of *de novo* DSA was associated with a decreased risk of CLAD or death (HR: 0.36, *p* < 0.01) (16). Deferring treatment until the patient clinically developed AMR was associated with an increased risk of CLAD or death (HR: 3.00, *p* < 0.01) (16). Early treatment was defined as preemptive antibody-directed therapy (including intravenous immune globulin, rituximab, plasma exchange, proteasome inhibitor and/or steroids) based only on the positive DSA (16). In fact, treating molecular AMR as defined by DSAs with elevation in other biomarkers like the dd-cfDNA may improve outcomes, and this is an area of active research. Studies of DSAs in lung transplant recipients vary widely in terms of lack of standardization of DSA testing, including difference in intervals at which DSAs are routinely monitored, particularly longer term. The interpretation of histologic AMR varies by institution, and sampling bias during transbronchial biopsy may limit the identification of AMR or C4d positivity on tissue samples. Generally, data is derived from retrospective or cohort studies, and randomized clinical trials are not available to guide assessment or treatment approaches.

**Table 1:** DSA studies in in lung transplantation.

Study	Study design	Sample size (N)	Testing utilized	Interval for testing	Outcome	Hazard ratio (95% confidence interval)
Roux et al. (12)	Prospective	209	HLA-Ab using LABScreen single antigen bead assay	Pre-LTx and postoperatively at days 1, 7, 21, and 30; months 2-6, 9, and 12; and then every 6 months	10.7% (22/206) had DSA + AMR	
					AMR was associated with CLAD in DSA+/AMR patients	8.7 (3.08–24.63)
					AMR was associated with graft loss in DSA+/AMR patients	7.56 (3.72–15.36)
Morrell et al. (13)	Prospective	445	Combination panel-reactive antibodies (PRA) by enzyme-linked immunosorbent assay and Luminex single-antigen bead assay	Pre-LTx and every 2–3 months for 2 years after transplantation in combination	13% (58/445) had *de novo* DSA	
					*de novo* DSA was associated with bronchiolitis obliterans syndrome (BOS)	6.59 (4.53–9.59)
					*de novo* DSA was associated with high grade (2 and above) BOS	5.76 (3.48–9.52)
Tikkanen et al. (14)	Prospective	320	Initial PRA testing pre-tx using solid phase microsphere technology on Luminex platform; in PRA greater than 0%, single-antigen bead testing was performed	Pre-tx and quarterly while on the wait list and at 2 weeks, 6 weeks, 3, 6, 9, 12, 18, and 24 months post transplantation	47% (161/320) patients with *de novo* DSA	
					*de novo* DSA was associated with CLAD	2.04 (1.13–3.69)
Le Pavec et al. (15)	Prospective	134	HLA typing using polymerase chain reaction sequence-specific primer at transplantation; if indicated using Luminex assays post transplantation	Before LTx and at day 7, months 1, 3, 6, and 12 post transplantation	61% (82/134) developed *de novo* DSA	
					Higher mean fluorescence intensity (MFI) of DSA levels were associated with CLAD	2.83 (1.42–5.67)
					Highest MFI of DSA was associated with mortality	2.71 (1.34–5.47)
Keller et al. (16)	Retrospective	445	Single antigen bead testing using LABScreen assay	Pre-Ltx and on day 7, 14, and months 1, 3, 6, 9, 12, 18 and 24 post transplantation	33% (145/445) developed *de novo* DSA	
					Early *de novo* DSA was associated with CLAD or death	0.36 (0.17–0.76)
					Delayed treatment until clinical AMR was associated with CLAD or death	3.00 (1.46–6.18)

DSA characteristics may also provide prognostic value. Patients with AMR have increased frequency of anti-HLA DQ-specific DSA and increased sum mean fluorescence intensity compared to patients without AMR (17). Persistent DSA and DQ-specific DSAs are associated with shorter time to chronic lung allograft dysfunction (CLAD) and decreased CLAD-free survival (18). Patients who developed C1q + DSAs also had shorter time to CLAD, and those with multiple DSAs had decreased CLAD-free survival (18). Further, HLA mismatch may have prognostic value in the development of primary graft dysfunction and the severity. Using high-resolution HLA matching, a study in 59 lung transplant recipients found that as the number of HLA antigen mismatch increased, including allele-level mismatch, the severity of primary graft dysfunction also increased (19). Higher HLA-DQ mismatch grade was significantly associated with severe primary graft dysfunction (19). In a retrospective cohort analysis of 128 lung transplant patients, HLA compatibility scores were calculated for B-cell epitopes, T-cell epitopes, and missing self-induced NK cell activation (20). Higher HLA compatibility scores for B-cell and T-cell epitopes were associated with more rapidly developing anti-HLA-DQ antibodies, and the HLA compatibility score of B-cell epitopes for HLA-DQ was significantly associated with worse survival (20).

### Non-HLA antibodies

Non-HLA antibodies have also been associated with increased risk of allograft dysfunction. These are alloantibodies directed against polymorphic antigens that vary between the donor and the recipient and autoantibodies (21). These self-antigens can develop against various receptors including collagen V, angiotensin type 1 receptor, and endothelin type A receptor (21). The exposure to these self-antigens is associated ultimately with loss of peripheral tolerance, which may lead to allograft dysfunction and rejection (21). In lung transplant patients with AMR without DSAs, non-HLA-positive antibodies are significantly higher than in patients without AMR (22). Non-HLA antibodies are also associated with an increased risk of CLAD (23), and the presence of DSA and non-HLA antibodies concurrently increases the CLAD risk further (23). In other solid organ transplantation, non-HLA antibody detection is typically performed through cell-based crossmatching assays or antigen detection methods such as enzyme-linked immunosorbent assay use, but genome-wide analyses and protein microarrays are also available (24). Nevertheless, screening for non-HLA antibodies is still not routinely performed in clinical practice at this time, and further efforts must be made to understand the impact of non-HLA antibodies in larger cohorts and standardize approach to testing (24).

### Dd-cfDNA

dd-cfDNA is a noninvasive measure of nucleosomes that are released into the bloodstream as cfDNA, which occur during graft injury, regardless of the underlying cause (25). These dd-cfDNA are markers of cell apoptosis and necrosis (26). Quantification of dd-cfDNA analyzes single-nucleotide polymorphisms to distinguish between donor and recipient molecules (27). Once the single-nucleotide polymorphisms are identified as related to the donor or recipient, the amount of cfDNA that exists is amplified with whole genome sequencing, quantitative polymerase chain reaction, or targeted sequencing, and typically, the percent dd-cfDNA over total cfDNA is reported, though some assays may report the absolute amount of dd-cfDNA as well (26). A retrospective case series (28) and prospective observational cohort study (29) of kidney transplant recipients suggests that absolute quantification of dd-cfDNA is better in discriminating biopsy-proven rejection compared to relative dd-cfDNA levels. However, a larger, prospective, biopsy-matched study of 367 kidney transplant patients suggests that using a combination of dd-cfDNA fraction (percent of total cfDNA) and quantity (genomic copies/ml), rather than either alone, is better to detect rejection, with a 73.5% sensitivity and 80.8% specificity (30). Use of dd-cfDNA in other solid organ transplantation has more evidence, particularly in kidney transplantation. In kidney transplantation, the European Society of Organ Transplantation recommends dd-cfDNA measurement in patients with allograft dysfunction to exclude rejection, particularly AMR, with a moderate strength based on a moderate quality of evidence (31). In heart transplantation, the 2023 International Society of Heart and Lung Transplant guidelines for the care of heart transplant recipients include the use of GEP (AlloMap® test), a plasma-based test for 11 genes associated with immune activation and inflammation (5), for surveillance of rejection (4).

## Natural progression of dd-cfDNA

The natural progression of dd-cfDNA is a stepwise decay over time in stable lung transplant recipients (32, 33). These levels are higher than those observed in heart or kidney transplant recipients. During the first two weeks after transplantation, dd-cfDNA levels increase due to ischemic-reperfusion injury (median 6.36%) (32). These levels stabilize thereafter but increase again during acute rejection (7.81%) and respiratory infections (9.14%) (32). Average dd-cfDNA levels vary over the first three months post-transplantation and follow three patterns of decay, divided into tertiles (34). Immediately after the transplant, all groups had high dd-cfDNA (34). Subjects in the lowest tertile had a rapid decline to a low level within one month of transplantation (34). Those in the middle tertile had a slow decline initially but, by three months, reached a stable level comparable to the lowest tertile (34). Subjects in the higher tertile showed a slower decay with persistent elevations in dd-cfDNA levels compared to the lower and middle tertile groups (34). An average dd-cfDNA increase of 1% was associated with a 1.4-fold increased risk of allograft failure (34). Those with higher tertile levels had a 6.6-fold increased risk of developing allograft failure compared to those in the low tertile, with a median time to developing allograft failure of 25 months compared to 45 months in the low tertile (34).

Several studies suggest lower baseline dd-cfDNA levels in single-lung recipients compared to double-lung recipients (32, 35). Dd-cfDNA level in one cohort of stable patients was 2.8% in single-lung recipients, 6.2% in double-lung recipients, and 13.3% in combined heart-lung recipients (32). In another cohort, dd-cfDNA levels were 0.15% in single-lung recipients compared to 0.46% in double-lung recipients (35). Discrepancies between the two fractions reported in these two studies stem from a difference in the method by which dd-cfDNA is measured in the varying assays. In acute rejection too, median dd-cfDNA levels vary, with median levels of 1.06% in single-lung recipients and 1.78% in double-lung recipients (35). The optimal thresholds of dd-cfDNA for the detection of acute rejection was 0.54% in single lung transplant and 1.1% in double-lung transplant recipients based on one study of 220 patients (35). The heterogeneity in the dd-cfDNA assays and reporting presents a challenge to the interpretation of the literature. However, in general, clinicians can expect a higher dd-cfDNA value in double-lung transplant recipients compared to single-lung recipients, though the difference in relative or absolute assay values is unclear.

### dd-cfDNA for determining presence of rejection

While the use of biomarker data to identify rejection in transplant recipients is promising, the evidence for the use of dd-cfDNA as a plasma biomarker of graft injury in lung transplantation is limited to retrospective and prospective cohort studies (Table 2). The most robust data comes from two cohorts, the single center Genome Transplant Dynamics study and the multicenter Genomic Research Alliance for Transplant (GRAfT) study of dd-cfDNA (34). Dd-cfDNA is inversely correlated with forced expiratory volume at one second (FEV1) (*R* = −0.26) (32). The level of dd-cfDNA that indicates graft injury varies from study to study in the literature, as do the sensitivities, specificities, positive predictive values, and negative predictive values (Table 3). Overall, most studies in the literature of lung transplantation recipients suggest an optimal dd-cfDNA cut-off varying between 0.85% and 1% (36–38, 39). When using a cut-off of ≥1% dd-cfDNA, sensitivities vary from 59.9 to 89.1%, specificities from 82.9 to 87.7%, positive predictive values of 43.4%–51.9%, and negative predictive values of 91.0%–96.5% (36, 38). Based on a study of 38 lung transplant recipients, using a threshold dd-cfDNA percent of 0.85% was associated with a sensitivity of 55.6%, specificity of 75.8%, a positive predictive value of 43.4%, and a negative predictive value of 83.6% to detect any allograft injury (37). The heterogeneity in these studies is important to note. The assays utilized to measure dd-cfDNA in these studies were varied, and it is difficult to know the comparison between assays. Further, not all cohorts performed transbronchial biopsies to confirm a diagnosis of ACR. Thus, an incorrect clinical diagnosis of ACR or a false negative biopsy may introduce bias.

**Table 2:** dd-cfDNA studies in lung transplantation.

Study	Study type	Sample size (N)	Outcome	Result	*p*-value
Agbor-Enoh et al. (34)	Prospective	106	Highest tertile average dd-cfDNA over 3 months was associated with allograft failure	Hazard ratio (95% Confidence Interval) 6.6 (1.6–19.9)	0.007
Sorbini et al. (32)	Prospective	30	dd-cfDNA levels were elevated during acute rejection	Mean 7.81% ± 12.7% (vs. 2.18% ± 3.26%)	<0.0001
			dd-cfDNA levels were elevated during respiratory tract infections	Mean 9.14 ± 15.59% (vs. 2.18% ± 3.26%)	0.0004
Keller et al. (35)	Prospective	221	dd-cfDNA levels were lower in single vs. double lung transplants	Median 0.15% vs. 0.46%, respectively	<0.01
			dd-cfDNA levels were higher in acute rejection for single lung transplant	Median 1.06% (vs. 0.15%)	0.05
			dd-cfDNA levels were higher in acute rejection for double lung	Median 1.78% (vs. 0.46%)	0.05
Keller et al. (36)	Retrospective	175	dd-cfDNA was higher in patients with acute allograft dysfunction vs. stable patients	Median 1.7% (vs. 0.35%)	<0.001
Khush et al. (37)	Prospective	38	dd-cfDNA was elevated in acute cellular rejection vs. stable patients	Median 0.91% (vs. 0.38%)	0.02
			dd-cfDNA was elevated in CLAD vs. stable patients	Median 2.06% (vs. 0.38%)	0.02
Rosenheck et (38)	Prospective	103	dd-cfDNA fraction was higher in acute cellular rejection vs. stable patients	Median 1.43% (vs. 0.46%)	0.000005
			dd-cfDNA fraction was higher in antibody-mediated rejection vs. stable patients	Median 2.50% (vs. 0.46%)	0.00002
			dd-cfDNA fraction was higher in infection vs. stable patients	Median 0.74% (vs. 0.46%)	0.02
			dd-cfDNA fraction was higher in CLAD vs. stable patients	Median 1.60% (vs. 0.46%)	0.00014
Ju et al. (39)	Retrospective	188	dd-cfDNA levels in rejection vs. stable patients	Median 1.34% (vs. 0.69%)	<0.001
			dd-cfDNA levels in infection vs. stable patients	Median 0.72% (vs. 0.69%)	<0.001
Agbor-Enoh et al. (41)	Prospective	157	Antibody mediated rejection was associated with higher dd-cfDNA	5.4% (vs. 1.1%)	<0.001
Sayah et al. (40)	Prospective	69	dd-cfDNA was elevated in acute cellular rejection vs. stable patients	Median 1.52% (vs. 0.485%)	0.026

**Table 3:** dd-cfDNA performance based on varying cut-off values.

Study	Study type	Sample size (N)	TBBx	dd-cfDNA assay	dd-cfDNA cutoff	Sensitivity	Specificity	PPV	NPV
Keller et al. (36)	Retrospective	175 lung recipients	Performed in some	Assay using next generation sequencing (CareDx)	≥1%	73.9%	87.7%	43.4%	96.5%
Khush et al. (37)	Retrospective	107 samples from 38 lung recipients	Performed	Assay using next generation sequencing (Allosure®)	0.85%	55.6%	75.8%	43.3%	83.6%
Rosenheck et al. (38)	Prospective	195 samples in 103 lung recipients	Performed	Assay using next-generation sequencing using Prospera test (Natera, Inc, Austin, TX)	≥1%	89.1%	82.9%	51.9%	97.3%
Ju et al. (39)	Retrospective	188 (lung or heart-lung recipients)	Performed if patient's condition allowed	Assay using next-generation sequencing (AlloDx Biotech, Co, Ltd)	0.89%	98.21%	82.58%	70.5%	99.1%

Abbreviations: NPV, negative predictive value; PPV, positive predictive value.

The mean baseline dd-cfDNA levels in stable patients also vary. In general, evidence suggests that dd-cfDNA levels are higher in acute rejection compared to stable patients without rejection. In a retrospective study of 188 lung and heart-lung transplant recipients, patients with rejection had higher levels of dd-cfDNA (median level 1.34%) compared with stable patients (median level 0.69%, *p* < 0.001) (39). A multicenter, retrospective study of 175 patients similarly found that dd-cfDNA levels were higher in patients with acute lung allograft dysfunction, defined as a composite of acute rejection and infection, with a median level of 1.7% compared with a median level of 0.35% in stable patients, *p* < 0.001 (36). In a study utilizing archived biorepository plasma samples, dd-cfDNA levels were elevated (1.06%) in the aggregate cohort of rejection [ACR—including A1 ACR, AMR, and bronchiolitis obliterans syndrome (BOS)], compared to dd-cfDNA levels in stable patients (0.38%) (37). Dd-cfDNA levels in the subtypes of acute rejection are inconsistent in the literature. A biorepository study of 69 lung transplant patients identified dd-cfDNA of 1.52% in patients with ACR compared to 0.485% in stable patents, though the diagnosis of ACR in these patients was predominantly A2B0 and A1B2r rejection, so higher grade rejection is not represented in this data (40). Prospectively collected data from 195 samples in 103 patients evaluated dd-cfDNA levels in four clinical-pathologic diagnoses of rejection (ACR, AMR, CLAD/neutrophil responsive allograft dysfunction, isolated lymphocytic bronchiolitis, and infection) and demonstrated statistically significant differences in median dd-cfDNA fraction among the groups (38). Patients identified to have ACR, AMR, or infection had higher median dd-cfDNA levels compared to stable patients (38). The median dd-cfDNA level was higher for ACR (1.43%), AMR (2.50%), infection (0.74%), and CLAD/neutrophil-responsive allograft dysfunction (1.6%) compared to stable patients (38). Of note, in this study, ACR and AMR were both classified as acute rejection (38). A study of the GRAfT and GTD cohorts utilizing 2016 International Society of Heart and Lung Transplantation (ISHLT) consensus criteria to adjudicate AMR and the ISHLT histopathologic criteria to adjudicate found that levels of dd-cfDNA are higher in patients with AMR (5.4%) compared with ACR (1.1%) (41). Dd-cfDNA was similarly found to be elevated with AMR, with median levels of 1.34%, compared with stable patients (0.38%), in a study utilizing 107 archived biorepository plasma samples from 38 patients, though this small sample size study did not reach statistical significance (37). Dd-cfDNA was able to identify AMR a median of 2.8 months before a clinical diagnosis of AMR was possible (41). In CLAD, too, data suggests elevated dd-cfDNA, with a median dd-cfDNA of 2.06% (37).

### dd-cfDNA and allograft infection

Differentiating infection from stable patients or acute rejection using dd-cfDNA is also difficult and adds uncertainty when utilizing dd-cfDNA in post-lung transplant monitoring. There was no statistically significant difference in dd-cfDNA levels in patients with allograft infection (median 0.39%) compared to stable patients in one study of 38 patients (37). A retrospective review of 188 lung transplant recipients attempted to utilize a combination of dd-cfDNA and next-generation sequencing for pathogen detection to differentiate between rejection and infection in patients who presented with new onset pulmonary complication. An elevated dd-cfDNA, combined with negative next-generation sequencing for pathogen results, was strongly indicative of rejection, with a sensitivity of 98.21%, specificity of 94.7%, positive predictive value of 88.7%, and negative predictive value of 99.2% (39). In contrast, elevated dd-cfDNA alone without next-generation sequencing for pathogen detection had a 98.21% sensitivity, 82.58% specificity, 70.5% positive predictive value, and 99.1% negative predictive value for diagnosing rejection (39). Patients with rejection had higher levels of dd-cfDNA, with a median level of 1.34%, compared to those with infection (median 0.72%, *p* < 0.001) (39). In this cohort, dd-cfDNA levels were significantly increased during infection compared to in stable patients (median 0.69%, *p* < 0.001) (39). Patients with CMV infection had significantly higher levels of dd-cfDNA compared to those with no infection (*p* < 0.001) (39).

### Prognostic and clinical use of dd-cfDNA

Extreme elevations of dd-cfDNA may provide prognostic value. A multicenter prospective cohort study of 328 lung transplant recipients evaluated dd-cfDNA and found that extreme molecular injury, defined as extreme elevation in dd-cfDNA in the upper quartile range (≥5%) of all patients with acute rejection after 45 days post-transplant, was associated with increased risk of severe CLAD or death (HR: 2.78, *p* = 0.012) (42). The time at which there was first evidence of this extreme molecular injury was a significant predictor of the likelihood of CLAD or death (AUC = 0.856) (42).

The use of dd-cfDNA may reduce the need for routine bronchoscopies. and one study suggested an 82.1% reduction in bronchoscopies performed compared to expected (36).

Based on the existing body of evidence, the 2024 European Society for Organ Transplantation Consensus Statement weakly recommends the use of dd-cfDNA to diagnose clinical and subclinical acute rejection compared to standard diagnostic methods, based on a low level of evidence (43). The group also weakly recommends the use of dd-cfDNA to diagnose infection of and as a reliable marker to stratify prognosis for CLAD, based on very low levels of evidence (43). Notably, groups recommend weakly against the use of dd-cfDNA as a therapeutic marker to monitor treatment response for acute rejection or infection, based on very low evidence (43). Further evidence is still needed to identify whether fraction or absolute dd-cfDNA levels are more relevant in lung transplant recipients (43).

### Next-generation sequencing for pathogens

Plasma microbial cell-free DNA sequencing uses next-generation sequencing to detect microbial cfDNA in the bloodstream that may not be identified by culture-based methods (44). The use of such next-generation sequencing tests for pathogens has been shown to increase diagnostic yield in immunocompromised patients (specifically, patients with hematologic malignancy who underwent hematopoietic cell transplantation (44). These tests have not been specifically validated in lung transplant recipient populations. Moreover, while the next-generation sequencing library is created and compared to an existing library of over 1,000 pathogens, this test has the potential to identify organisms that may not be pathogenic (5), and results must be interpreted within the clinical context.

### Markers of immunity

The immune cell function (ICF) assay measures the adenosine triphosphate levels that are released by CD4+ T cells (45). This assay can be used to evaluate the cellular immune response in lung transplant recipients and potentially identify patients who may be at increased risk of developing rejection or infection (45). Median ICF values have been noted to be significantly different in cytomegalovirus (CMV) disease, viral infections, and bacterial infections compared with stable patients (46). ICF assay levels are lower in infected lung transplant recipients compared to non-infected patients (47). In retrospective studies of ICF assay in heart transplant recipients, levels <300 ng/ml identified patients at risk of CMV infection but noted that ICF assay levels were not significantly different in fungal or bacterial infections (48). TTV testing involves monitoring of TTV levels, which are small single-stranded DNA viruses that are ubiquitous in the majority of humans (49). Levels of viral replication are known to correlate with immune response (49, 50). TTV levels increase in response to immunosuppressive therapy, suggesting increased viral replication with the reduction of immunocompetence, and higher levels of posttransplant TTV are associated with increased microbial infections (49). Monitoring of Epstein–Barr virus (EBV) DNA loads has also been utilized as a measure of immunosuppression in lung transplant recipients and may help guide adjustments in immunosuppression (51).

## Discussion

The use of routine bronchoscopy at specified intervals after luntr transplantation has been the standard of care for the identification of subclinical rejection. The availability of biomarkers holds significant promise for the future, though the literature remains largely retrospective, prospectively collected on biorepository samples, or with sample sizes. In general, there is consistentcy in the literature that supports the use of dd-cfDNA, combined with clinical evaluation and monitoring of other biomarkers, such as DSAs, for identification of rejection. However, the exact values of dd-cfDNA which indicate rejection, differentiation of the etiology of dd-cfDNA elevation, and differentiation of infection from acute rejection from chronic rejection, relies largely on the clinical context. Future research is needed to evaluate the diagnostic accuracy of dd-cfDNA in diagnosing acute rejection compared with the current gold standard of transbronchial biopsy. Additionally, the role of newer biomarkers such as non-HLA antigens and TTV in clinical practice is yet to be determine, and further real-world application is needed.

### Our current practice

**Figure 2:**
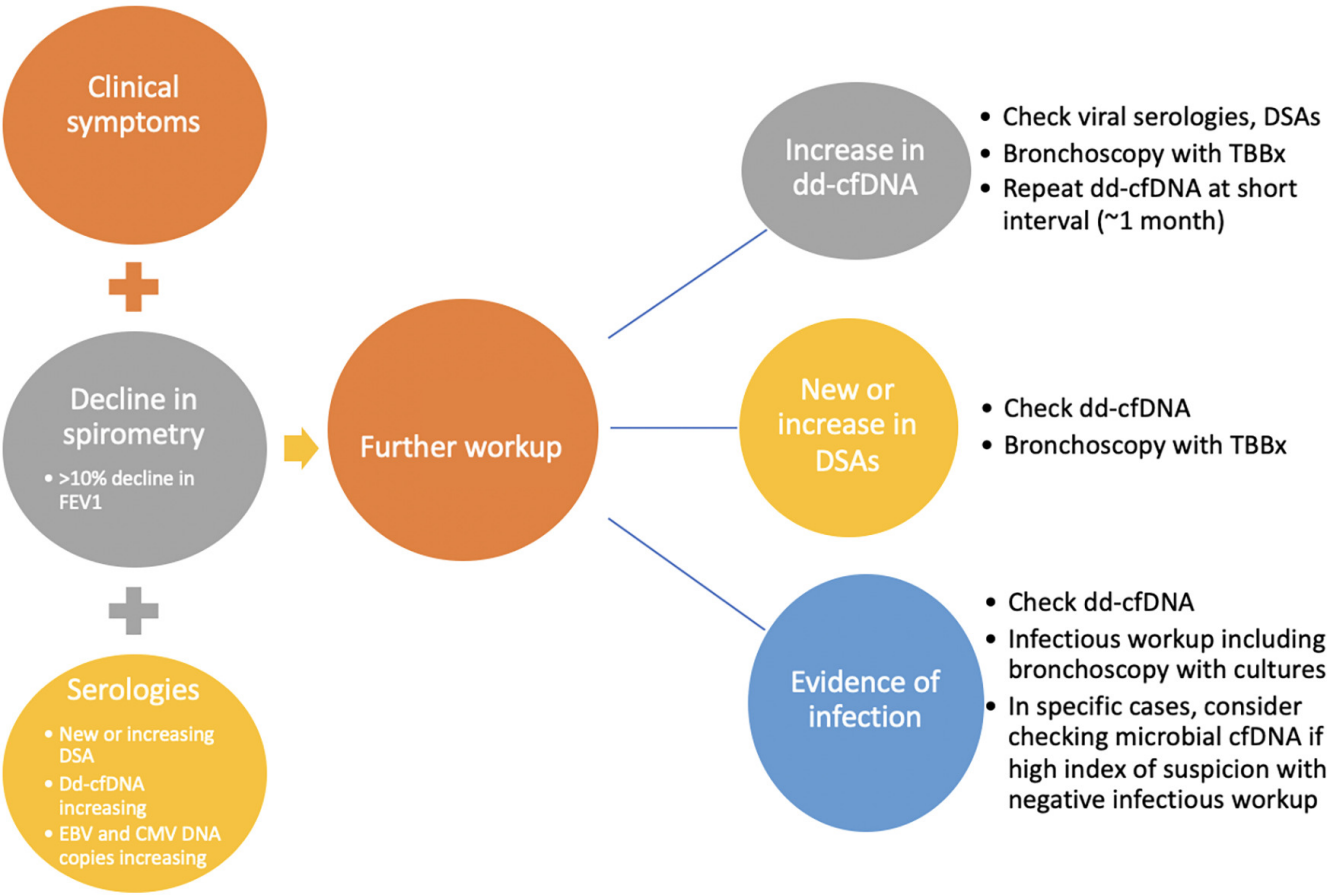
Approach to evaluation for allograft dysfunction. Allograft function is evaluated routinely by monitoring clinical symptoms, spirometry, and serologies. Evidence of clinical symptoms of rejection or infection, decline in spirometry (specifically decline in FEV1 > 10%, and evidence of serologic changes including new or increasing DSAs, increasing dd-cfDNA, or increasing EBV or CMV DNA copies prompts further investigation. Further investigation includes more inclusive infectious workup, bronchoscopy with transbronchial biopsy (TBBx), repeat of dd-cfDNA at a short interval, checking of immune cell function (ICF) assay, and consideration for evaluation of microbial cfDNA, in the right clinical context. Adapted with permission from “Overview of the potential genetic and epigenetic approaches associated with the analysis of cell free DNA” by Michael B. Keller, Temesgen E. Andargie and Sean Agbor-Enoh, licensed under CC BY 4.0.

As noted above, evidence for the use of biomarkers in lung transplantation recipients is still accumulating, and there are no clear guidelines yet. However, we are gathering more experience through the use of biomarkers in clinical practice. We share our current approach, summarized in Figure 2. It should be noted that the use of the biomarkers is often individualized and requires careful review of clinical, spirometric, radiologic, and functional data.

**Figure 3:**
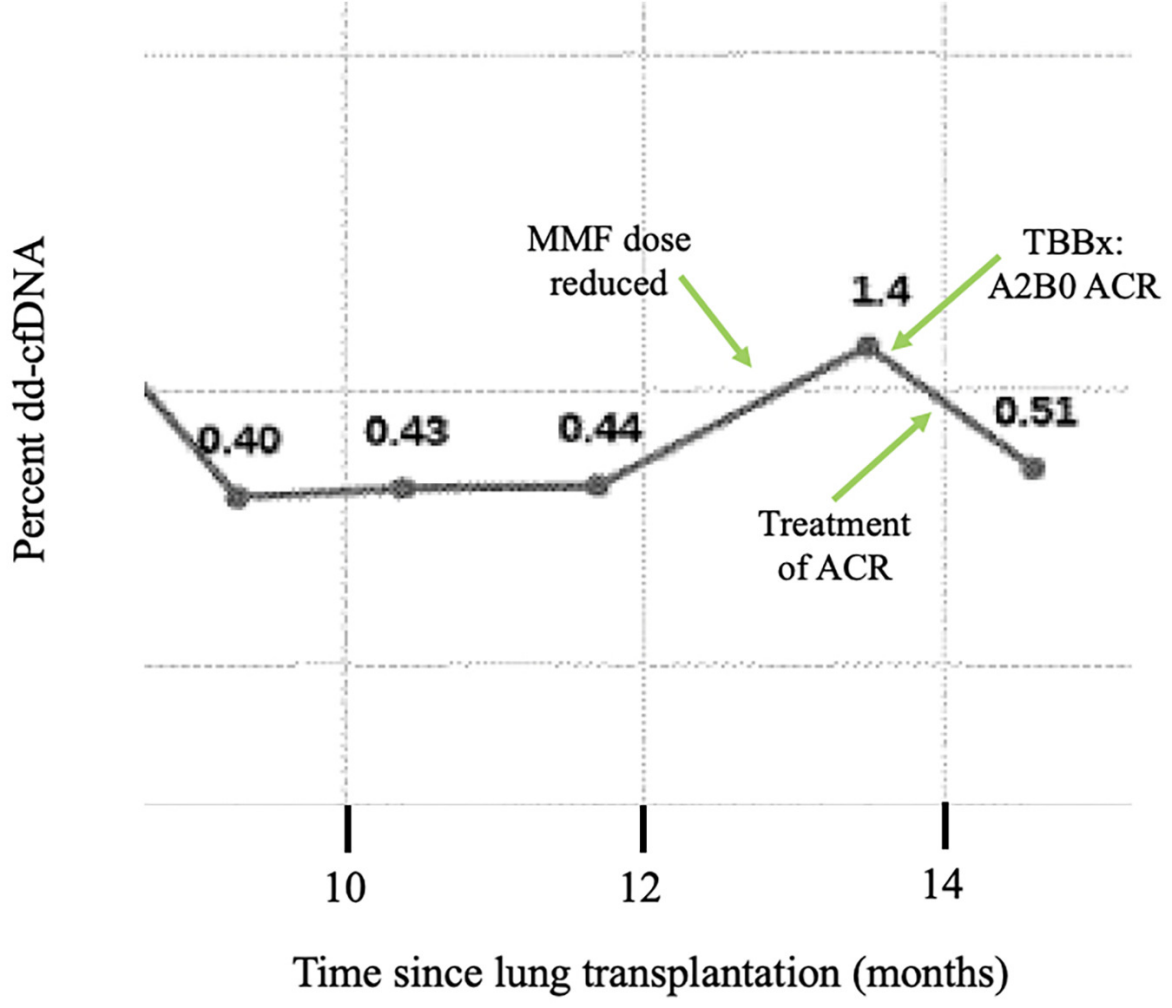
Example case: trends of dd-cfDNA in ACR. The dd-cfDNA percent is shown across time in a 42-year-old bilateral lung transplant recipient for cystic fibrosis. The patient had stable dd-cfDNA until around 12 months post-transplantation. Due to a positive anal pap smear, his mycophenylate mofetil dose was reduced. Around month 13, routine dd-cfDNA demonstrated increase compared to prior. A transbronchial biopsy demonstrated A2B0 acute cellular rejection, and the patient was treated with steroids. Subsequently, repeat dd-cfDNA declined. Abbreviations: ACR, acute cellular rejection; MMF, mycophenylate mofetil.

As is standard practice in many transplant programs, DSA evaluation is performed routinely before, during, and after the transplantation period. Our practice is to monitor dd-cf-DNA monthly for the first year and quarterly thereafter. We measure DSAs at month 1 and then every 3 months for the first year and if negative, every 6 months thereafter; patients with known DSAs get them assessed every 3 months. Extreme changes in DSA or trends toward elevation provide a clue to the presence of AMR, oftentimes before clinical evidence. Routine use of dd-cfDNA beginning one month after transplantation allows for early identification of ACR or acute rejection, or it may coincide with clinical manifestations and culture evidence of infection which requires treatment. We do not routinely employ one value as a dd-cfDNA cutoff for rejection. Rather, the trend in dd-cfDNA allows for heightened suspicion of rejection. Our practice is to perform bronchoscopy with transbronchial biopsy at one month and three months post transplantation, with additional bronchoscopies and transbronchial biopsies performed based on changes in dd-cfDNA, DSAs, spirometry, or clinical symptoms. In patients with clinical evidence of infection, such as recurrent fevers, increased secretions on bronchoscopy, etc., we selectively send next-generation microbial cf-DNA sequencing, though we do not use this solely to diagnose infections in the vast majority of our patients. Routine monitoring of ICF assay and EBV DNA copies after transplantation also allows for early detection of reduction in immune cell function and consideration for increased risks for certain infections, particularly CMV or other viral infections. While the ICF assay may be an older test, we utilize the test during management of immune suppression medications post-transplantation and while weaning immune suppression. Our approach can be illustrated in an example case of a 42-year-old man who underwent bilateral lung transplantation for cystic fibrosis about 13 months prior to presentation. Routine DSA monitoring remained negative. Dd-cfDNA was also being monitored every 2–3 months and were consistently between 0.40 and 0.44% (Figure 3). The patient was initially on triple immunosuppression with tacrolimus, prednisone, and mycophenolate mofetil during this time. Due to an anal pap smear demonstrating atypical cells, the mycophenolate dose was reduced. The patient's routine ICF assay values, which were 518 ng/ml ATP and 674 ng/ml ATP (consistent with high immune cell response), decreased to 277 ng/ml ATP (moderate immune cell response). Routine follow up of dd-cfDNA about one month after mycophenolate dose reduction demonstrated an increase to 1.4%. The patient underwent transbronchial biopsy, and pathologic review revealed A2B0 rejection. The patient received intravenous steroids, and repeat dd-cfDNA subsequently demonstrated reduction to 0.51%, closer to baseline, and the patient did not require a repeat biopsy to confirm resolution. Utilizing a combination of biomarkers, the diagnosis of ACR was suggested prior to any clinical evidence of rejection. Thus, on a case-by-case basis, we can combine biomarkers with a careful history, physical examination, and routine pulmonary function testing to monitor for acute rejection in the post-lung transplantation patient.

## Conclusion

Allograft dysfunction remains a large contributor to morbidity and mortality in lung transplant recipients. Current methods of monitoring for rejection rely on clinical presentation of symptoms or decline in pulmonary function tests which may not be present, or which may present later in the course of rejection. Biomarkers have the potential to allow for early and less invasive diagnosis of rejection, but further studies are required to fully elucidate their exact application in this patient population. Methods to differentiate between rejection and infection are required. Utilization of current biomarkers, namely DSA, dd-cfDNA, measures of immune suppression, and sometimes plasma microbial cell-free DNA next-generation sequencing, in combination with clinical evaluation and histology assessment, may help in the diagnosis of allograft dysfunction including different forms of rejection as well as infection.
